# Proteomic Identification of an Endogenous Synaptic SUMOylome in the Developing Rat Brain

**DOI:** 10.3389/fnmol.2021.780535

**Published:** 2021-11-23

**Authors:** Marie Pronot, Félicie Kieffer, Anne-Sophie Gay, Delphine Debayle, Raphaël Forquet, Gwénola Poupon, Lenka Schorova, Stéphane Martin, Carole Gwizdek

**Affiliations:** ^1^Centre National de la Recherche Scientifique, Institut de Pharmacologie Moléculaire et Cellulaire, Université Côte d’Azur, Nice, France; ^2^Institut National de la Santé Et de la Recherche Médicale, Centre National de la Recherche Scientifique, Institut de Pharmacologie Moléculaire et Cellulaire, Université Côte d’Azur, Nice, France

**Keywords:** synapse, post-translational modification, SUMO, SUMOylome, proteomics

## Abstract

Synapses are highly specialized structures that interconnect neurons to form functional networks dedicated to neuronal communication. During brain development, synapses undergo activity-dependent rearrangements leading to both structural and functional changes. Many molecular processes are involved in this regulation, including post-translational modifications by the Small Ubiquitin-like MOdifier SUMO. To get a wider view of the panel of endogenous synaptic SUMO-modified proteins in the mammalian brain, we combined subcellular fractionation of rat brains at the post-natal day 14 with denaturing immunoprecipitation using SUMO2/3 antibodies and tandem mass spectrometry analysis. Our screening identified 803 candidate SUMO2/3 targets, which represents about 18% of the synaptic proteome. Our dataset includes neurotransmitter receptors, transporters, adhesion molecules, scaffolding proteins as well as vesicular trafficking and cytoskeleton-associated proteins, defining SUMO2/3 as a central regulator of the synaptic organization and function.

## Introduction

A functional neuronal network relies on the coordinated organization of billions of highly specified contact points called synapses that interconnect neurons and allow communication in the mammalian brain. During the brain development, synapses undergo constant activity-dependent protein content rearrangements leading to both structural and functional changes. Many processes are involved in this modulation, including proteins, mRNAs and organelle transports along axons and dendrites as well as synaptic vesicular trafficking, synaptodendritic exchanges, local translation and protein degradation. Post-translational modifications (PTM) such as SUMOylation regulate protein function as well as the shaping of macromolecular complexes and participate in those processes.

SUMOylation consists in the covalent but reversible attachment of one or more SUMO peptides (around 100 amino acids, i.e., 11 kDa) on lysine residues of target proteins. SUMO conjugation is achieved by the sole E2 SUMO-conjugating enzyme Ubc9. Substrate recognition by Ubc9 is either direct or mediated by bridging proteins called E3 ligases. There are three SUMO paralogs (SUMO 1, 2, and 3) in the mammalian brain. SUMO2 and SUMO3 are nearly identical except three additional N-terminal residues in the SUMO3 sequence and are generally referred to SUMO2/3. SUMO1 shares around 50% identity with SUMO2/3 and even though SUMO1 and SUMO2/3 have an overlapping set of targets and functions, they differ in their subcellular abundance and their properties to form poly-SUMO chains (Cappadocia and Lima, 2018; Chang and Yeh, 2020). SUMOylation is readily reversible upon the activity of specific deSUMOylation enzymes called SENPs.

At the molecular level, protein SUMOylation can inhibit interactions by promoting a steric hindrance, or contribute to new protein-protein interactions by providing an additional binding site for partners presenting SUMO-Interacting Motifs (SIMs) (Cappadocia and Lima, 2018; Chang and Yeh, 2020). The consequences of SUMOylation are diverse. For instance, it can regulate the activity or the subcellular localization of some target proteins and/or enhance or inhibit protein degradation *via* its crosstalk with the Ubiquitin system. SUMOylation can also impact protein solubility and/or aggregation (Janer et al., 2010; Henley et al., 2021) and acts as a spatiotemporal shaping of protein–protein interactions within macromolecular complexes (Matunis et al., 2006).

The SUMOylation process was initially characterized in the nucleus, where SUMO substrates are far more abundant. However, it is now clear that SUMOylation also plays important extranuclear roles, for instance in mitochondrial fusion/fission (Harder et al., 2004), cytoskeleton organization (Alonso et al., 2015) or in cellular trafficking (Wasik and Filipek, 2014). In the brain, both the SUMO machinery expression and protein SUMOylation profiles vary according to brain areas (Akiyama et al., 2018), cell types (Maruyama et al., 2018) and sub-cellular compartments (Loriol et al., 2012; Colnaghi et al., 2019, 2020; Schorova et al., 2019) and evolves with brain maturation and neuronal activity (Watanabe et al., 2008; Andreou and Tavernarakis, 2010; Craig and Henley, 2012; Loriol et al., 2012, 2014; Ficulle et al., 2018; Josa-Prado et al., 2019; Schorova et al., 2019). As expected, the nuclear compartment of neuronal cells is highly enriched in SUMO enzymes and substrates (Loriol et al., 2012; Colnaghi et al., 2019). In synapses, the levels of SUMOylated proteins are much lower, with the highest levels occurring during the synaptogenesis period in early life (Loriol et al., 2012). The presence of synaptic SUMOylated proteins has been questioned (Daniel et al., 2017) despite the multiple reports demonstrating the functional and physiological impact of specific SUMOylated proteins at synapses (Lee et al., 2013; Schorova and Martin, 2016; Liu et al., 2017; Marcelli et al., 2017; Wilkinson et al., 2017; Henley et al., 2021). In line with these reports, recent work using super resolution microscopy confirmed the presence of SUMO1, SUMO2/3, Ubc9, and SENPs in both pre- and post-synaptic compartments (Colnaghi et al., 2019, 2020).

Of particular interest is also the role of SUMOylation in the regulation of several synaptic proteins (Schorova and Martin, 2016; Henley et al., 2021) involved in spine formation (Khayachi et al., 2018), neuronal excitability (Plant et al., 2011), presynaptic neurotransmitter release (Girach et al., 2013; Tang et al., 2015) and synaptic communication and plasticity (Martin et al., 2007; Loriol et al., 2014; Choi et al., 2016). In addition, neuronal activity often acts as a trigger to promote SUMOylation (Loriol et al., 2013, 2014; Schorova et al., 2019) leading to a fine tuning of the synaptic function.

Although the repertoire of identified SUMO substrates at synapses is regularly expanding (Henley et al., 2021), a broader view of the SUMO-modified proteins at the mammalian synapse is still missing. Given the peak of synaptic SUMO2/3-ylated proteins during the synaptogenesis period, we performed subcellular fractionation on PND14 rat brains to isolate the synaptic compartments, followed by denaturing immunoprecipitations with specific SUMO2/3 antibodies to enrich the preparation in SUMOylated substrates and finally, mass spectrometry analysis to identify the isolated SUMO2/3-modified proteins at the mammalian synapse. We then validated some SUMO-modified proteins among the 803 identified SUMO2/3 targets. This unique dataset represents about 18% of the synaptic proteome and includes neurotransmitter receptors, transporters, adhesion molecules, scaffolding proteins as well as vesicular trafficking and cytoskeleton-associated proteins, revealing a central regulatory function of the mammalian synapse by the SUMOylation process.

## Materials and Methods

### Rat Strain

Wistar rats were exclusively from a commercial source (Janvier, St Berthevin, France). All animals were handled and treated in accordance with the ARRIVE Guidelines. Animals had free access to water and food. Lightning was controlled as a 12 h light and dark cycle and the temperature maintained at 23 ± 1°C. For brain retrieval, PND14 pups were euthanized by decapitation according to a protocol approved by the Animal Care and Ethics Committee and forebrains were immediately excised and used.

### Synaptosomal Preparation

Synaptosomal preparation was adapted from previously published protocols (Loriol et al., 2014). Fresh forebrains of four PND14 rats were homogenized in 26 mL of ice-cold Sucrose Buffer (0.32 M Sucrose, 10 mM Tris–Hcl pH 7.4, complete EDTA-free protease inhibitor cocktail Roche) with 20 mM NEM to prevent proteins from deSUMOylation using a 30 mL glass-Teflon homogenizer with 12 gentle strokes. The homogenate (total fraction, Tot) was centrifuged at 1,000 *g* for 5 min at room temperature (RT) in a JA4.2 rotor (Beckman). The supernatant (cytoplasmic fraction, Cyt) was then layered by 2 mL on the top of a four-step Percoll-sucrose density gradient (2 mL of 20% Percoll, 2 mL of 10% Percoll, 2 mL of 6% Percoll, and 2 mL of 2% Percoll in Sucrose Buffer) and centrifuged at 18,000 rpm for 10 min at 4°C in a SW41Ti rotor (Beckman). Synaptosomal fraction was recovered at the 10–20% interface Percoll layers. This fraction was then washed in 10 mL of HEPES Buffer (5 mM HEPES, 140 mM NaCl, 3 mM KCl, 1.2 mM MgSO4, 1.2 mM CaCl2, 1 mM NaH2PO4, 5 mM NaHCO3, 10 mM Glucose, and complete EDTA-free protease inhibitor cocktail Roche, 10 mM NEM) and centrifuged at 10,000 *g* for 10 min in JA25.5 rotor (Beckman). The synaptosomal pellet was resuspended in Denaturing Lysis Buffer (20 mM Sodium Phosphate buffer pH7.4, 150 mM NaCl, 5 mM EDTA, 5 mM EGTA, 1% Triton X-100, 0.5% DOC, 1% SDS, complete EDTA-free protease inhibitor cocktail Roche, 10 mM NEM), supplemented with 50 mM DTT and boiled for 10 min at 95°C. Protein concentration was determined using standard Bradford assay.

### Antibodies Immobilization on Protein-G Sepharose Beads

For IP beads preparation, Protein-G Sepharose beads (Sigma, P3296) were washed three times and incubated in PBS for 45 min at RT with SUMO-2/3 mouse monoclonal antibody (Clone 12F3, Cytoskeleton) or mouse IgG antibody as a negative control. After two washes in 20 mM Sodium Phosphate buffer (NaPi) pH7 and two washes in Triethanolamine Buffer (200 mM Triethanolamine, pH 8.2), beads were incubated for 1 h at RT in 30 mM dimethylpimelidate (DMP) freshly resuspended in Triethanolamine Buffer to crosslink the antibodies to the beads. The crosslinking step was stopped by resuspension of the beads in 50 mM Tris–HCl pH8. Finally, the beads were washed once with 200 mM Glycine pH2.9 and twice with 20 mM NaPi pH8.

### Immunoprecipitation Experiments

The denaturing immunoprecipitation protocol was adapted from Becker et al., 2013. Briefly, the denaturated synaptosomal fractions were diluted in Dilution Buffer (20 mM NaPi pH7.4, 150 mM NaCl, 5 mM EDTA, 5 mM EGTA, 1% Triton X-100, 0.5% DOC, and complete EDTA-free protease inhibitor cocktail Roche, 10 mM NEM) to obtain a final concentration of 0.1% SDS. The lysate was centrifuged for 10 min at 20,000 *g* at 4°C. 8 mg of proteins, corresponding to synaptosomes isolated from ten PND14 rat brains, were incubated with 160 μg of SUMO-2/3 mouse monoclonal antibody, or mouse IgG antibody as a negative control immobilized on 60 μL of Protein-G Sepharose beads, overnight at 4°C under soft rotation. Beads were washed three times for 5 min at 4°C in Wash Buffer (20 mM NaPi pH7.4, 150 mM NaCl, 5 mM EDTA, 5 mM EGTA, 1% Triton X-100, 0.5% DOC, 0.1% SDS, and complete EDTA-free protease inhibitor cocktail Roche). Beads were additionally washed in Wash Buffer containing 500 mM NaCl for 30 min at 37°C. Proteins bound to the beads were then eluted three times for 30 min at 37°C with 200 μL of Wash Buffer supplemented with 0.5 mg/mL of SUMO2 peptide (CQIRFRFDGQPINE). Eluates were pooled and subjected to TCA precipitation prior to quality controls and mass spectrometry analysis.

*SUMO2/3-ylated proteins immunoprecipitation from total brain extracts* were performed as described above with few modifications: Beads were coated after antibody immobilization in Coating Buffer (PBS 1X, BSA 5 mg/mL, Dextran 40 kDa, 5 mg/mL, and Gelatin 1 mg/mL). Proteins bound to the beads were eluted in 2X Laemmli buffer + 5% β-mercaptoethanol.

#### Target Immunoprecipitation

For the validation of SUMO targets, synaptosomal fractions were denaturated in Denaturation buffer (20 mM NaPi pH7.4, 150 mM NaCl, 5 mM EDTA, 5 mM EGTA, 1% Triton X-100, 1% SDS, complete EDTA-free protease inhibitor cocktail Roche, 10 mM NEM) for 15 min at 56°C. The denaturated synaptosomal fraction was diluted in Dilution Buffer to obtain a final concentration of 0.1% SDS. Lysates were centrifuged for 15 min at 20,000 *g* at 4°C. 800 μg of proteins were incubated with immobilized anti-Ubc9 or not immobilized anti-SynGAP or anti-Flotillin-1 on coated Protein-G Sepharose beads, overnight at 4°C under rotation. The corresponding IgG antibody was used as a negative control. The beads were then washed 3 times at 4°C in Wash Buffer. Proteins were eluted in Laemmli Buffer supplemented with 5% β-mercaptoethanol.

### Mass Spectrometry Analysis

#### Sample Preparation

Proteins from immunoprecipitation eluates were separated on SDS-PAGE on Bis-Tris gradient gel (4–20%, Mini-PROTEAN^®^, BioRad) and colored by Coomassie staining (Imperial blue). Each lane was manually excised into bands. Proteins contained into gel slices were reduced/alkylated and digested by a treatment with DTT/IAA and trypsin. Tryptic peptides were isolated by successive extractions in 1% formic acid (FA), in water and in acetonitrile (ACN). Peptides extracts were concentrated under vacuum and solubilized in 15 μL of aqueous 0.1% formic acid. The resulting peptides mix were then subjected to LC-MS/MS analysis.

#### NanoHPLC-Q-Exactive Plus Analysis

Peptide separations were carried out on a nanoHPLC (ultimate 3000, Thermo Fisher Scientific). 5 μL of peptidic solution was injected and concentrated on a μ-Precolumn Cartridge Acclaim PepMap 100 C18 (i.d. 5 mM, 5 μm, 100 Å, Thermo Fisher Scientific) at a flow rate of 10 μL/min and using solvent containing H_2_O/ACN/FA 98%/2%/0.1%. Next, peptides separation was performed on a 75 μm i.d. x 500 mM (3 μm, 100 Å) Acclaim PepMap 100 C18 column (Thermo Fisher Scientific) at a flow rate of 200 nL/min. Solvent systems were: (A) 100% water, 0.1%FA, (B) 100% acetonitrile, 0.08% FA. The following gradient was used *t* = 0 min 4% B; *t* = 3 min 4%B; *t* = 170 min, 35% B; *t* = 172 min, and 90% B; *t* = 180 min 90% B (temperature set at 35°C). The nanoHPLC was coupled *via* a nanoelectrospray ionization source to a Hybrid Quadrupole-Orbitrap High Resolution Mass Spectrometer (Thermo Fisher Scientific). MS spectra were acquired at a resolution of 70 000 (200 m/z) with a scan range of 150-1800 m/z, an AGC target value of 5e5 and a maximum injection time of 50 ms. 10 most intense precursor ions were selected and isolated with a window of 2 m/z and fragmented by HCD (Higher energy C-Trap Dissociation) with normalized collision energy (NCE) of 27. MS/MS spectra were acquired in the ion trap at a resolution of 17 500 (200 m/z) with an AGC target value of 2e5 and a maximum injection time of 100 ms.

#### Protein Identification

Data were reprocessed using Proteome Discoverer 2.2 equipped with Sequest HT. Files were searched against the Swissprot Rattus Norvegicus Reviewed and Unreviewed FASTA database. A mass accuracy of ± 10 ppm was used to precursor ions and 0.02 Da for product ions. Enzyme specificity was fixed to trypsin with two missed cleavages allowed. Because of previous chemical modification, carbamidomethylation of cysteines was set as a fixed modification and only oxidation of methionine was considered as dynamic modification. Reverses decoy databases were included for all searches to estimate false discovery rates, and filtered using Percolator algorithm with a 1% FDR.

### SDS-PAGE and Western Blotting

Samples from the SUMO2/3 immunoprecipitations and the associated IgG negative control were resolved by SDS-PAGE followed by silver staining (Silver Quest Kit; Invitrogen) for quality control or Coomassie staining (Imperial blue) prior to in gel trypsin digestion and mass spectrometry analysis. For immunoblotting, proteins separated by SDS-PAGE were transferred onto nitrocellulose membrane (BioTrace NT, Pall Corporation) and immunoblotted using the following primary antibodies at the indicated dilution: mouse anti-SUMO2/3 (clone 12F3, Cytoskeleton, ASM23) 1/1000, rabbit polyclonal anti-SUMO2/3 (Dr. Guillaume Bossis, IGMM, France) 1/750, mouse anti-SUMO1 (clone 21C7, DSHB) 1.5 μg/mL, mouse anti-Synaptotagmin (Stressgen, SYA-148) 1/2000, rabbit anti-PSD95 (Millipore, AB9708) 1/2000, rabbit anti-Homer (Synaptic System, 160003) 1/1000, mouse anti-CoxIV (Abcam, ab14744) 1/2000, mouse anti-SOD2 (Santa Cruz, sc-137254) 1/2000, mouse anti-Calnexin (Santa Cruz, sc-23954), rabbit anti-GM130 (BD Biosciences, 610823) 1/500, mouse anti-Nopp140 (Santa Cruz, sc-374033) 1/700, mouse anti-Fibrillarin (Invitrogen, MA3-16771) 1/2000, rabbit anti-Histone H4 (Active Motif, 39269) 1/2000, mouse anti-Ubc9 (BD Biosciences, 610749) 1/250, rabbit anti-SynGAP (Synaptic System, 157002) 1/1000; and mouse anti-Flotillin-1 (BD Biosciences, 610820) 1/500. Primary antibodies were revealed using the appropriate horseradish peroxidase (HRP)-conjugated secondary antibodies (GE healthcare) or True Blot (Rockland, Tebu-Bio). Proteins were then identified using Immobilon Western (Millipore), SuperSignal Femto chemiluminescence (Thermo Scientific) or Western Lightning Ultra (Perkin Helmer) chemiluminescent solutions and images acquired on a Fusion FX7 system (Vilber Lourmat).

### Bioinformatics Analysis

To compare proteome datasets, protein identifiers were converted to Entrez Gene identifiers using Uniprot, DAVID and db2db conversion tools. When rat datasets were compared to human or mouse datasets, human or mouse homologs to rat proteins were identified using the DRSC Integrative Ortholog Prediction Tool or alternatively Blast. Gene Ontology (GO) terms enrichment analyses were performed using clusterProfiler (ontology level 2) on the web interface ProteoRE (Nguyen et al., 2019). KEGG and Reactome pathways enrichment analysis were performed using DAVID. Enrichment analysis using the synapse specific database SynGO (Koopmans et al., 2019) were performed against the “brain expressed” background, setting medium stringency and second level terms as labels for Cellular Component representation and top-levels terms as labels for Biological Pathways representation. Disease annotations were conducted with the ToppGene portal on the human homologs of the rat protein dataset. Network analysis were performed using the STRING web tool (v. 11.0) combined to the Cytoscape application (v.3.8.2) implemented by the StringApp plug-in. Details for enrichment analyses are available in Supplementary Table 6.

## Results

### Strategy to Identify Endogenous Synaptic SUMO2/3-Modified Proteins

**FIGURE 1:**
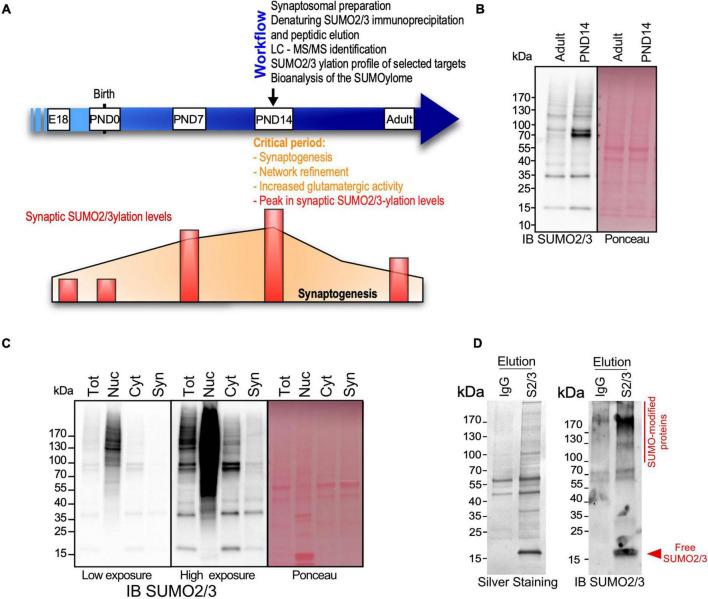
Identification of the synaptic SUMO2/3-ylome. **(A)** Workflow to identify the synaptic SUMO2/3-ylome. PND14 rat brains, presenting intense synaptogenesis, neurotransmission and high levels of SUMO2/3-ylation, were subjected to biochemical subcellular fractionation to prepare synaptosomes. Denaturing immunoprecipitations were performed on the synaptosomal fractions using specific SUMO2/3 antibodies and peptidic elution. Isolated proteins were then identified by LC-MS/MS. The SUMO2/3-ylation profile of several selected targets was analyzed by biochemical approaches and the biological significance of the synaptic SUMO2/3-ylome was brought out by bio-informatics analysis. **(B)** Immunoblotting of SUMO2/3-ylation levels in PND14 and adult brain for SUMO2/3 (left panel). Ponceau-S staining was used to detect total proteins (right panel). **(C)** SUMO2/3-ylation levels detected by immunoblotting on 20 μg of proteins from indicated subcellular fractions. After low-speed centrifugation of the total brain lysate (Tot), the supernatant corresponding to the cytoplasmic fraction (Cyt) was loaded on sucrose percoll gradients to isolate the synaptosomal fraction (Syn) (Loriol et al., 2014) and the nuclear fraction (Nuc) in the pellet was purified on sucrose cushions (Boisvert et al., 2010). **(D)** Ten percent of the SUMO2/3 12F3 (S2/3) or control IgG immunoprecipitated proteins were subjected to silver staining (left panel) or SUMO2/3 immunoblotting (right panel).

The expression levels of SUMO-modified substrates as well as the components of the SUMOylation machinery are developmentally and spatially regulated in the mammalian brain, with a peak of SUMO2/3-ylated proteins at the post-natal day (PND) 14 (Loriol et al., 2012) during the synaptogenesis period (Semple et al., 2013; Figures 1A,B). PND14 rat brains were thus subjected to biochemical fractionation to purify the synaptosomal fraction (Figure 1C). The quality of the fractionation was assessed by immunoblotting using antibodies against specific markers for different subcellular compartments (Supplementary Figure 1A). As expected, the synaptosomal fraction was highly enriched in synaptic markers such as PSD95, Synaptotagmin and Homer1 compared to the other fractions. Synaptosomes also contain mitochondria and some endoplasmic reticulum. In line with this, the respective markers for mitochondria and endoplasmic reticulum, CoxIV and Calnexin, were also detected in the synaptosomal fractions. Importantly, synaptosomes were totally devoid of nuclear markers confirming the high quality of the fractionation process (Supplementary Figure 1A).

Four independent synaptosomal preparations from PND14 rat brains were subjected to proteomics analysis. We established a list of 4379 proteins detected in at least 3 of the 4 preparations (Supplementary Table 1). This protein list was then used as our synaptic proteome reference. More than 88% of the proteins listed were found in at least three previously published synaptic proteomes and 97% were detected at least once as synaptic components (Supplementary Table 2). Gene Ontology (GO) enrichment analysis showed that among the top 20 for enriched GO Cellular Components terms, ten classes are associated to synapses whereas the other terms are linked to mitochondria and vesicular structures. Consistently, the top 20 for enriched GO Biological Processes terms revealed the involvement of the identified proteins in synapse organization, vesicle organization and trafficking, mitochondria organization as well as nucleotide metabolic processes (Supplementary Figures 1B,C). Altogether, these data confirmed the enrichment of the samples in synaptic components further highlighting the quality of the synaptosomal purification.

Previous proteomic studies on brain SUMOylation were performed using adult and/or whole brain extracts (Tirard et al., 2012; Yang et al., 2014; Matsuzaki et al., 2015; Hendriks et al., 2018; Stankova et al., 2018) and consequently, identified a high proportion of nuclear proteins as neuronal SUMO targets. To elaborate the most efficient protocol to assess synaptic SUMO2/3-ylation, we compared the levels of SUMO2/3-ylated proteins between synaptosomes and other subcellular fractions of the brain (Figure 1C). As expected, the SUMO2/3 immunoreactivity was maximum in the nuclear fraction, whereas synaptic SUMO2/3-ylation levels represented less than 1% of the total brain SUMO2/3-ylation. Considering the dynamic range of the Orbitrap Q-Exactive Plus mass spectrometer used in this study, it was absolutely necessary to enrich the preparations in synaptic material prior to isolate SUMO2/3-ylated proteins by denaturing immunoprecipitation (Becker et al., 2013) and identification by mass spectrometry.

To specifically isolate synaptic SUMO2/3-modified proteins, we performed immunoprecipitation using a commercially available mouse monoclonal SUMO2/3 antibody (Clone 12F3) that binds an epitope within the peptide CQIRFRFDGQPINE in SUMO2/3 (Becker et al., 2013; Supplementary Figure 2). This antibody specifically recognized proteins conjugated to SUMO2 but not conjugated to SUMO1 (Supplementary Figure 2A). Besides, peptide competition experiments monitored by dot blot using recombinant SUMO2 confirmed that free CQIRFRFDGQPINE peptides interfere between SUMO2 and the monoclonal SUMO2/3 antibody (Supplementary Figure 2B). We then showed that SUMO2/3 antibodies are able to specifically immunoprecipitate SUMO2/3-ylated proteins from rat brain in denaturing conditions (Supplementary Figures 2C,D). We thus performed a SUMO2/3 immunoprecipitation combined with a peptidic elution (Becker et al., 2013) to purify SUMO2/3-ylated proteins from denatured rat synaptosomes. Solubilization in 1% SDS efficiently extracted proteins from synaptic membranes, in particular those embedded in detergent resistant compartments such as the post-synaptic density (Jordan et al., 2006). Given the low levels of SUMO2/3-modified proteins at synapses, we performed immunoprecipitation experiments on synaptosomal lysates purified from a total of 10 PND14 rat brains per point. To assess the quality of this process, 10% of the proteins isolated with SUMO2/3 antibodies or mouse IgG as negative control were separated on gradient SDS-PAGE and silver stained (Figure 1D, left panel) or immunoblotted for SUMO2/3 (Figure 1D, right panel). Eluates from SUMO2/3 antibody-mediated immunoprecipitation showed a clear enrichment in SUMOylated proteins compared to eluates from matching IgG controls confirming that this approach allows to efficiently isolate synaptic SUMO2/3-ylated proteins.

Four synaptic SUMO2/3 immunoprecipitation assays with their IgG controls were performed independently. After protein separation by SDS-PAGE and in gel Trypsin digestion, samples were analyzed by liquid chromatography coupled to tandem mass spectrometry (LC-MS/MS). MS data were processed using Proteome Discoverer 2.2 against the Swissprot Rattus Norvegicus database and only Master Proteins with a 1% False Discovery Rate (FDR) for both peptides and proteins were considered. Then, the list of synaptic SUMO2/3-ylated proteins was built using the following selection criteria: (i) for each SUMO2/3 immunoprecipitation experiment, proteins present in the respective IgG control were subtracted from the proteins identified in the SUMO2/3 immunoprecipitates and (ii) the proteins present in at least three of the four filtered lists were selected as candidate SUMO2/3 targets. This finally led to a list of 803 synaptic SUMO2/3-ylated proteins with 291 (36%) present in all four experiments (Supplementary Table 3).

### Insights Into the Synaptic SUMO2/3-ylome

**FIGURE 2:**
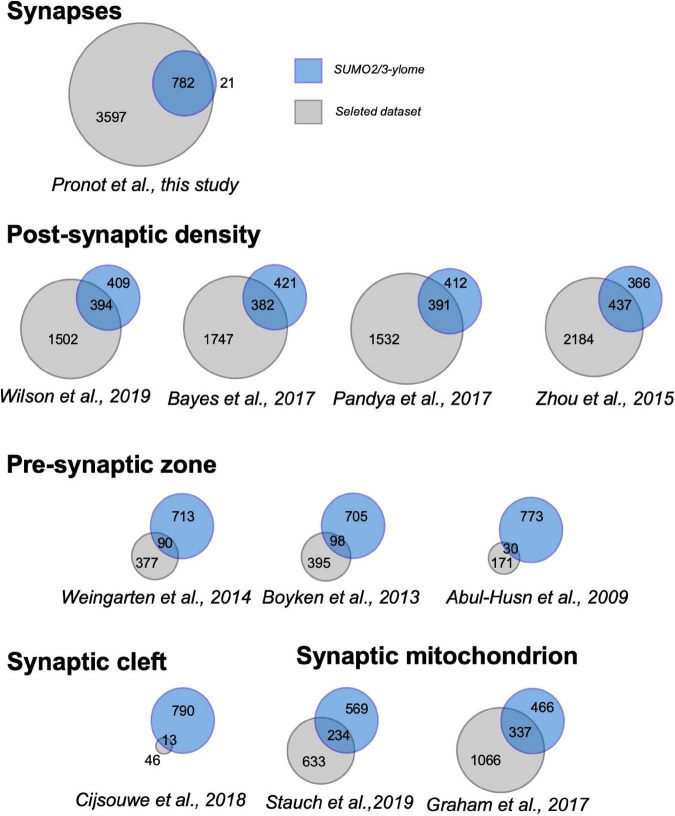
Venn diagrams showing overlap between the synaptic SUMO2/3-ylome and the indicated proteomes. Numbers indicate the number of proteins specifically present in the present study (blue), in the referenced previous studies (gray) and at the intersection of the protein sets. Details in the Supplementary Table 4.

To analyze the relevance of the synaptic SUMO2/3-ylome, we first compared the list of SUMO2/3-ylated target proteins with previously published proteomes obtained from synaptosomes or subspecific components of synaptosomal fractions (Figure 2 and Supplementary Table 4). We showed that more than 97% of the SUMO2/3-ylome were detected in at least five synaptic datasets with about 40% of the candidate SUMO2/3 target proteins associated with the Post-Synaptic Density, on average over the different selected PSD proteomes (Supplementary Table 4). Thus, the list of SUMO2/3-ylated candidate proteins identified largely overlapped with previously characterized synaptosomal protein datasets, demonstrating that the SUMO2/3-ylome is directly connected to the synaptic compartment.

Noteworthy, our dataset presented many proteins found to be SUMOylated in previous target specific studies such as mitochondrial proteins (DJ-1/Park7 (Guerra de Souza et al., 2016), Sirt3 (He et al., 2020), constituents or regulatory factors of the cytoskeleton (β-Actin, α-Tubulin, RhoGDI, and Septins) (Alonso et al., 2015; Feng et al., 2021), signaling molecules (RAS (Choi et al., 2018), β-catenin (Karami et al., 2017), CYLD (Kobayashi et al., 2015)), proteins involved in cellular trafficking (Flotillin-1 (Jang et al., 2019), EDH3 (Cabasso et al., 2015), and hnRNPA2B1 (Villarroya-Beltri et al., 2013)) as well as the well described SUMO-conjugating enzyme Ubc9 (Loriol et al., 2012; Hendriks et al., 2018), the PKC-α and PKC-ε (Zhao et al., 2020; Henley et al., 2021) or SOD1 (Fei et al., 2006).

**FIGURE 3:**
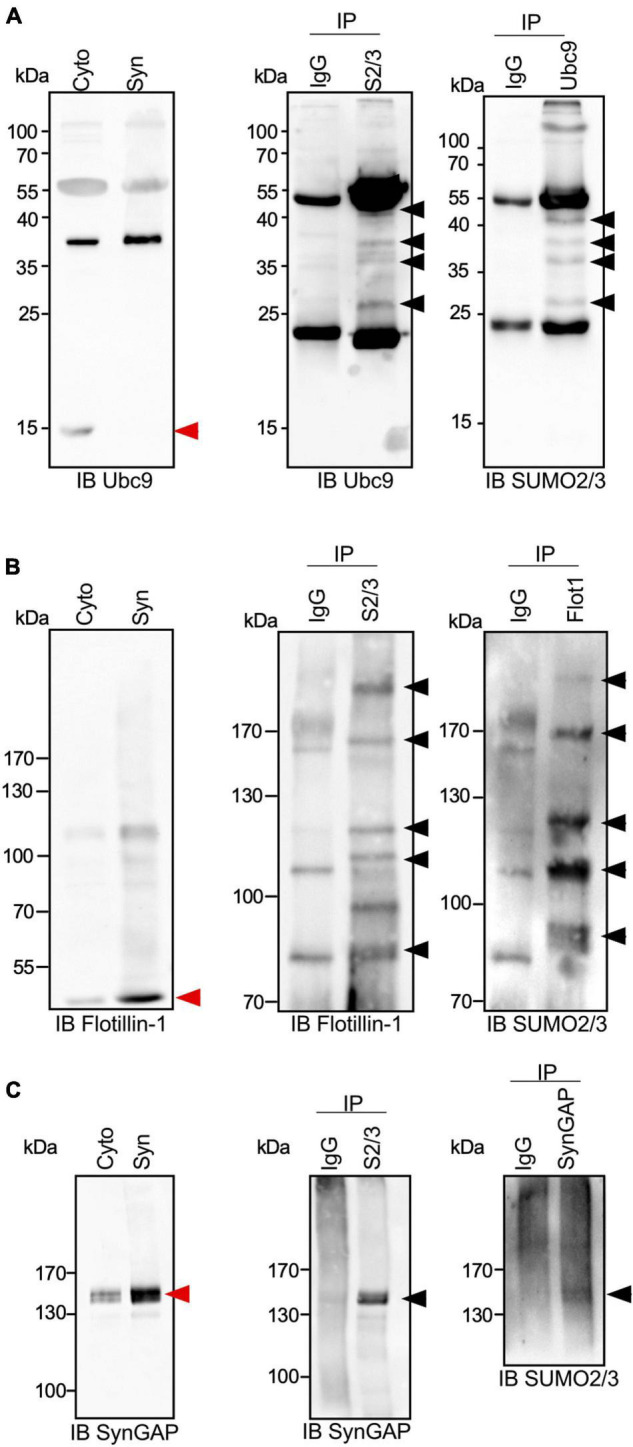
SUMO2/3-ylation profile of Ubc9, Flotillin-1 and SynGAP at synapses. **(A)** Immunoblot anti-Ubc9 of cytoplasmic and synaptic extracts from PND14 rat brains (left panel). Representative immunoblot using Ubc9 antibody of synaptic SUMO2/3 or control IgG immunoprecipitated proteins (middle panel; *n* = 3 independent experiments) and immunoblot using SUMO2/3 antibody of synaptic Ubc9 or control IgG immunoprecipitated (right panel; *n* = 3 independent experiments). Similarly to **(A)**, immunoblot of cytoplasmic and synaptic extracts from PND14 rat brains (left panel), synaptic SUMO2/3 or control IgG immunoprecipitated proteins (middle panel; *n* = 3 independent experiments) and the converse immunoprecipitation (right panel; *n* = 3 independent experiments) were done for Flotillin-1 **(B)** and SynGAP **(C)**. Black arrows indicate SUMO2/3-ylated forms of the different SUMO targets and red arrows indicate the unmodified forms.

To go deeper into the validation of the proteomic screen, we next verified the endogenous SUMOylation profile of a selection of candidate SUMO2/3 targets by immunoprecipitation/blotting with specific target antibodies. We first analyzed the modification of the SUMO conjugating enzyme Ubc9 that was previously reported to be SUMOylated in the brain by both SUMO1 and SUMO2/3 (Loriol et al., 2012; Hendriks et al., 2018). In PND14 synaptosomes, free Ubc9 is barely detected as a 18 kDa protein and appears mainly as a 38 kDa modified form (Figure 3A, left panel) that corresponds to a SUMO1-conjugated form (Loriol et al., 2012). Several SUMO-targeted lysines were identified in Ubc9 by proteomics or target specific approaches (Knipscheer et al., 2008; Loriol et al., 2012; Hendriks et al., 2018). Here, we showed at least four bands upper to the unmodified Ubc9 by immunoprecipitation assays on PND14 synaptosomal lysates using SUMO2/3 antibodies followed by anti-Ubc9 immunoblot (Figure 3A, middle panel). Similar results were obtained in converse experiments where Ubc9 immunoprecipitation was combined with SUMO2/3 immunoblotting (Figure 3A, right panel), thus confirming the modification of Ubc9 by SUMO2/3 at synapses.

Using a similar approach based on conversed and complementary immunoprecipitations, we analyzed the SUMO2/3-ylation profile of Flotillin-1/Reggie-2 (Figure 3B). Flotillin-1 is a multifunctional protein involved in signaling, actin cytoskeleton remodeling or receptor endocytosis/trafficking (Kwiatkowska et al., 2020). In the brain, it participates in synapse formation and neurite branching (Kwiatkowska et al., 2020). Flotillin-1 has been shown to be SUMOylated on several target lysines in cell lines (Hendriks et al., 2018) and in a cellular model of prostate cancer (Jang et al., 2019). Our data indicate that Flotillin-1 is also modified by SUMO2/3 at synapses, presenting multiple conjugated forms in line with the previous reports (Figure 3B).

We next examined the SUMO2/3-ylation of the Synaptic Ras-GTPase-activating protein SynGAP (Figure 3C). SynGAP is a downstream effector of NMDA receptors that tunes down the activity of Ras and Rap GTPases and impacts AMPA receptor trafficking within postsynaptic membranes (Jeyabalan and Clement, 2016). SynGAP is predominantly localized at the PSD where it interacts with the scaffolding protein PSD-95. Both the SUMO2/3 immunoprecipitation on synaptosomal extracts followed by SynGAP immunoblotting and its converse experiment allowed the detection of a 140–150 kDa SUMO2/3-ylated form of the protein (Figure 3C). This slight band shift corresponding to the SUMO-modified form of SynGAP may indicate that the protein is only modified by one SUMO2/3 moiety.

In addition to these biochemical validations, we compared our dataset to two previously published rodent brain SUMO2/3-ylomes (Yang et al., 2014; Hendriks et al., 2018). Thirty-six proteins of our synaptic SUMO2/3-ylome (i.e., 4.5%) were formerly shown to be modified by SUMO2/3 in the Central Nervous System (Supplementary Table 5). This narrow overlap is largely explained by the fact that these proteomic analyses were performed on whole adult brain extracts without any synaptic enrichments. Interestingly, an extended comparison with SUMOylomes obtained from 15 previous proteomics analysis in other cellular models indicates that 62% of our identified SUMO targets were also found in these studies. The overlap increased to 74% when alternative approaches were included (Supplementary Table 5). Altogether, these analyses further validated the SUMO immunoprecipitation approach on synaptosomal-enriched fractions from PND14 brains.

### SUMO2/3, a Central Regulator of the Synaptic Organization and Function

**FIGURE 4:**
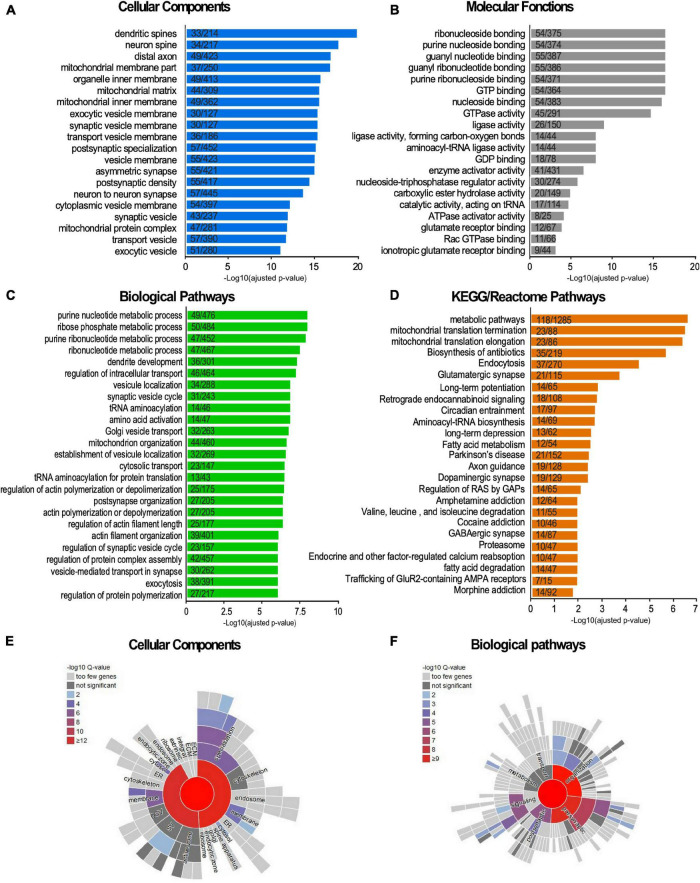
Terms enrichment analysis of the synaptic SUMO2/3-ylome according to the Gene Ontology, KEGG, Reactome and SynGO knowledge bases. **(A–D)** The list of the SUMO2/3-modified synaptic proteins was subjected to enrichment analysis for GO Cellular Components **(A)**, GO Molecular functions **(B)**, GO Biological Pathways terms **(C)**, for KEGG and Reactome pathways **(D)** against the rat proteome. Categories were classified according to the -Log10 of the adjusted *p*-value using the Benjamini- Hochberg method. The number of counts compared to the total number of hits in the category is indicated. The list of the SUMO2/3-modified synaptic proteins was subjected to enrichment analysis for Cellular Components **(E)** or Biological Pathways **(F)** using the SynGO synaptic component curator tool. Details are available in Supplementary Table 6.

We identified 803 SUMO2/3-modified protein candidates, which represents around 18% of our reference synaptic proteome (Figure 2 and Supplementary Table 4). To assess the biological meaning of this synaptic SUMO2/3-ylome, we performed Gene Ontology terms as well as KEGG and Reactome pathways enrichment analyses (Figure 4). Consistent with the clear overlaps with known synaptic proteomes and sub-proteomes (Figure 2 and Supplementary Table 4), the top 25 for enriched GO Cellular Components terms are connected to synapses, mitochondria and vesicles. Regarding GO Biological Processes or KEGG/Reactome pathways (Figures 4C,D), the synaptic SUMO2/3-modified proteome was enriched in proteins associated with neuronal development, organization and function, such as dendritic development (30 genes/proteins found in the SUMO2/3 dataset over 301 genes/proteins in the category), axon guidance (19/218), post-synapse organization (27/205), long-term potentiation (14/65) or depression (13/62), as well as glutamatergic (21/115), dopaminergic (19/129) and GABAergic synapses (14/87). In line with an enrichment in glutamatergic synapses, the synaptic SUMO2/3-ylome was also highlighted for the molecular function of glutamate receptor (12/67) and ionotropic glutamate receptor binding (9/44) (Figure 4B). Besides, vesicle-associated pathways as well as mitochondrial organization and processes, closely related to the synaptic function, clearly emerged in these analyses. Interestingly, the regulation of the actin cytoskeleton, which is involved in the shaping and intracellular trafficking at synapses as well as the proteasome, which participates in synaptic protein homeostasis, were also featured (Figures 4C,D). Cellular Components and Biological Processes enrichment analyses using the synapse specific annotation database SynGO (Koopmans et al., 2019) clearly corroborated the involvement of the synaptic SUMO2/3-ylome in synaptic organization, transmission and plasticity (Figures 4E,F). Last, enrichment analysis of the SUMO2/3-modified proteins dataset in KEGG pathways also highlight classes referring to brain disorders such as addictions and neurodegenerative diseases (Figure 4D).

**FIGURE 5:**
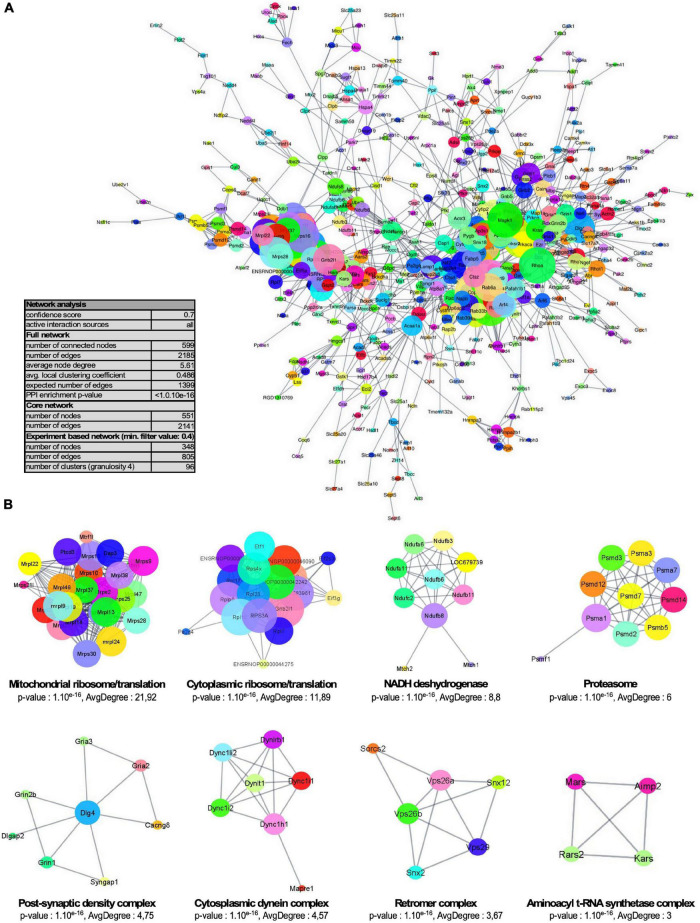
Network organization of the synaptic SUMO2/3-ylome. **(A)** Core cluster obtained by STRING network analysis of all identified SUMO2/3-ylated protein candidates, with a STRING interaction confidence of 0.7 or greater on all active interaction sources. **(B)** STRING network was imported into the Cytoscape application through the StringApp plug-in and further filtrated for experiments edges with a confidence of 0.4 or greater. The resulting sub-complex (346 components) was subjected to MCL clustering at granulosity 4, which resulted in 96 sub-complexes including eight clusters with an average degree superior or equal to three illustrated here. Node size reflects the average node degree within the network.

One of the main roles of SUMOylation is the modulation of protein-protein interactions. Therefore, proteins subjected to SUMOylation are highly interconnected (Hendriks et al., 2014). Besides, synaptic subdomains are organized around large dynamic protein networks. We thus tested the interconnectedness of the synaptic SUMO2/3-ylome using the STRING tool. As illustrated in Figure 5A, the synaptic SUMO2/3 targets assembled in a highly organized network. 74% of all identified proteins (599/803) were connected at high STRING confidence with an average node degree of 5.61, and 92% of these proteins (551/599) belonged to the core cluster. Using the Cytoscape resource, only experimentally validated interactions were extracted and the resulting network was submitted to cluster analysis (Figure 5B). Our settings highlight eight sub-complexes with an average node degree superior or equal to 3. The four prevalent clusters comprised numerous functionally related proteins from the cytoplasmic and mitochondrial ribosomes, the NADH dehydrogenase and the proteasome. Consistent with these findings, modification of the proteasomal subunits was robustly reported in different SUMO proteomes (Hendriks et al., 2014; Im and Chung, 2016; Pfammatter et al., 2018; Supplementary Table 5). In addition to the proteasome, ribosomes were also detected as one of the most dominant clusters in SUMOylome network analysis (Hendriks et al., 2014; Pfammatter et al., 2018) and many mitochondrial ribosomal proteins were detected as SUMO substrates in several proteomics studies (Supplementary Table 5). The other clusters were composed of proteins of the PSD, some aminoacyl t-RNA synthetases (ARS) associated with the ARS-interacting multi-functional protein two or some members of the cytoplasmic dynein complex as well as the retromer complex involved in intracellular trafficking.

**FIGURE 6:**
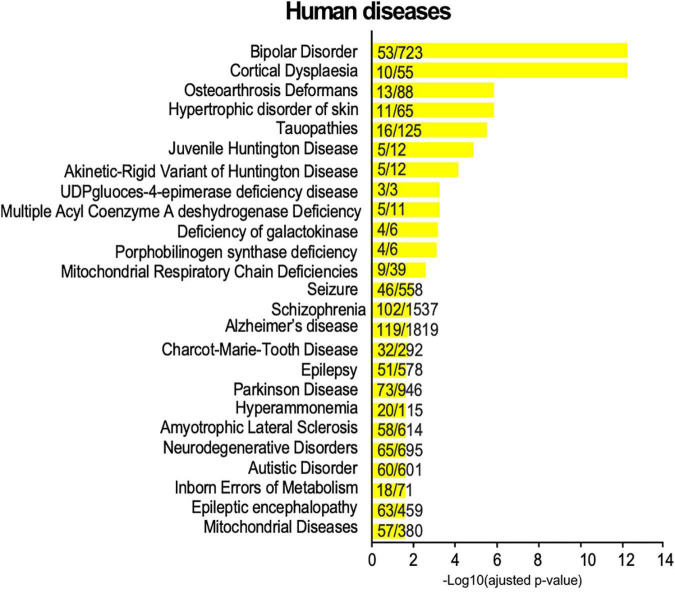
Disease annotation enrichment analysis of the synaptic SUMO2/3-ylome. Proteins from the SUMO2/3 dataset were analyzed for correlation with human diseases using ToppGene with the full gene set as background. Categories were classified according to the -Log10 of the adjusted *p*-value using the Benjamini-Hochberg method. The number of counts compared to the total number of hits in the category is indicated. Details are available in Supplementary Table 6.

In the above sections, we highlighted an enrichment in SUMO2/3-modified proteins dataset for KEGG pathways referring to addiction and neurodegenerative diseases (Figure 4D). To get a broader view of the connections between the synaptic SUMO2/3-ylome and human brain disorders, we interrogated diseases databases using the ToppGene web tool. We found that 318 proteins out of the 803, which corresponds to about 40% of the SUMO2/3-ylome, are connected with at least one brain disease (Supplementary Table 6). In the enrichment analysis, the first 25 hits displayed many neurodevelopmental, psychiatric or neurodegenerative disorders, including, but not restricted to, schizophrenia, autism, epilepsy, Alzheimer’s and Parkinson’s diseases (Figure 6).

## Discussion

During the formation of a functional neuronal network, synapses undergo massive molecular, structural and functional changes. Post-translational modifications dynamically control protein-protein interactions, regulating their functions and are thus, key determinants for these essential processes. In the present study, we identified 803 candidate SUMO2/3-modified proteins at the synapses of PND14 rat brains by a proteomic approach.

Surprisingly, earlier studies using the double-tagged His6-HA-SUMO1 knock-in mice concluded that SUMO1-ylated proteins are not present at synapses (Tirard et al., 2012; Daniel et al., 2017). Additional SUMOylation proteomic studies on rodent adult and/or whole brain extracts (Yang et al., 2014; Hendriks et al., 2018) have identified only 5% of the proteins reported in the current MS study (Supplementary Table 5). This low overlap can be explained first, by the absence of synaptic fractionation prior to enrichment in SUMO substrates and/or the use of adult brains where SUMO levels are much lower than in younger animals. Indeed, the main difficulties classically encountered in the identification of endogenous SUMOylated proteins are the reversible nature of the SUMO conjugation process and the low proportion of modified proteins for most SUMO substrates. In the case of synapses, the levels of SUMOylation are very low in comparison to the levels detected in other subcellular compartments. We estimate here that the nuclear fraction shows SUMOylation levels of more than one hundred-fold higher than in the synapse. In addition, the overall volume of the synaptic compartment is minimal compared to the rest of the cell. Synapses also present highly cohesive subdomains like the postsynaptic density, compromising the efficient biochemical protein extraction. To circumvent these difficulties, there is an absolute need to enrich biochemical preparations in synaptic proteins and use a substantial amount of starting materials. We thus purified SUMO2/3-ylated proteins by denaturing immunoprecipitation on synaptic preparations from 10 PND14 rat brains per point i.e., when the synaptic SUMO levels are at their maximum (Loriol et al., 2012), prior to the identification of SUMO2/3-ylated substrates by tandem mass spectrometry. These conditions should be considered as a gold standard to efficiently identify synaptic SUMO targets at the mammalian synapse. Among the 803 synaptic SUMO2/3 target proteins identified, we experimentally validated three of them, Ubc9, Flotillin-1 and SynGAP. Moreover, a bibliographic analysis revealed that many proteins of this dataset are found SUMOylated in previous target specific studies (Fei et al., 2006; Loriol et al., 2012; Villarroya-Beltri et al., 2013; Alonso et al., 2015; Cabasso et al., 2015; Kobayashi et al., 2015; Guerra de Souza et al., 2016; Karami et al., 2017; Choi et al., 2018; Hendriks et al., 2018; Jang et al., 2019; He et al., 2020; Zhao et al., 2020; Feng et al., 2021; Henley et al., 2021). Last, the comparison of our protein list to available SUMOylomes characterized by proteomics or by alternative approaches revealed that there is an overlap of more than 70% with the current dataset (Supplementary Table 5).

Surprisingly, our protein list contains a few proteins traditionally referenced as nuclear SUMO targets. This is the case for instance for RanGAP1, one of the most abundant and stable SUMO targets, predominantly associated to the nuclear envelope. Interestingly, RanGAP1 has been observed in the nucleus of neurons but localizes also in their axons and dendrites (Yudin et al., 2008; Mencarelli et al., 2018) where it may regulate the availability of factors involved in axonal growth or anterograde transport by controlling the GTP-bound state of the small GTPase Ran. The current list of proteins also contains Trim28/Kap1, a transcriptional repressor and a ubiquitin and SUMO ligase able to auto-SUMOylate (Cheng et al., 2014). Yet, Trim28/Kap1 has been shown to ubiquitinylate targets out of the nucleus such as the vacuolar protein sorting Vps34 (Yang et al., 2013) or the mitochondrial BCL2A1 (Lionnard et al., 2019). Consistently, both RanGAP1 and Trim28 have been referenced in previous synaptic datasets (Supplementary Table 2).

The network analysis of SUMO targets as well as the identification of the cellular compartments and the biological pathways enriched in our dataset revealed that many protein clusters are regulated by SUMO at the synapses including ribosomal and proteasomal protein complexes. These two machineries play an essential role in synaptic protein homeostasis, ribosomes by promoting local translation and the proteasome by driving protein degradation. Components of both complexes were frequently identified in SUMO proteomics analysis (Hendriks et al., 2014; Im and Chung, 2016; Pfammatter et al., 2018). However, the functional role of SUMO in the regulation of these complexes is still poorly defined. These protein assemblies, combining multivalent but dynamic interactions, are perfect candidates for protein-group SUMOylation (Jentsch and Psakhye, 2013). Indeed, collective SUMOylation of proteins also referred as ‘SUMO spray’ may stabilize and/or modulate these complex assemblies, SUMO acting as a glue to hold protein components together through intermolecular SUMO-SIM interactions. Given the size and complexity of the synaptic SUMO2/3-ylome, it is tempting to extend this ‘SUMO spray’ to both pre- and postsynaptic compartments. Protein-group SUMOylation could thus modulate and/or shape synaptic multimeric complexes. Nevertheless, it is not possible to assess the real-time SUMOylation states of multiple proteins in or out of synaptic complexes with the molecular tools currently available.

Several proteins located in the post-synaptic density have been identified as SUMOylated in this MS analysis. The PSD is composed of hundreds of different proteins including neurotransmitters receptors, scaffold proteins and many signaling molecules organized as thickly packed but highly dynamic interconnected assemblies creating a defined synaptic subdomain. Recent studies suggest that the assembly and activity-dependent modulation of the PSD composition involves liquid-liquid phase separation (LPPS) (Wu et al., 2020). LLPS governs the formation of many membraneless compartments or macromolecular structures such as the nucleolus, the PML bodies and stress or mRNA transport granules (Bratek-Skicki et al., 2020). Interestingly, increasing evidence point out a central role of SUMOylation in the composition of LLPS-based structures. One of the best-characterized examples is its role in the tuning of PML bodies formation (Bratek-Skicki et al., 2020). More recently, SUMO has been involved in the regulation of the LLPS-promoted SOP-2 bodies (Qu et al., 2020) and in stress granules assembly and disassembly (Marmor-Kollet et al., 2020). It is interesting to note that intrinsically disordered regions (IDR) that are key drivers to LLPS, are the preferential domains targeted by SUMO (Hendriks et al., 2017). At synapses, LLPS would be involved in the organization of the PSD notably through interactions between PSD95 and SynGAP (Wu et al., 2020). Since we showed that SynGAP is SUMO2/3-ylated at synapses, it would be of interest to examine whether its modification could interfere with its interaction with PSD95 and thus, participate in the regulation of the synaptic organization. LLPS may also play a role in the clustering of the reserve pool of synaptic vesicles through Synapsin 1-mediated interactions or *via* the formation of active zone complexes containing RIM1α and RIM-binding proteins (Wu et al., 2020). Strikingly, RIM1α and Synapsin 1 are known SUMO targets (Girach et al., 2013; Craig et al., 2015), Synapsin 1 being modified in its IDR at the lysine 687. Since the present dataset includes numerous proteins associated with the PSD as well as components of synaptic vesicles, we can easily speculate that SUMOylation of some of these proteins represents an effective way to modulate the dynamic formation and/or elimination of synaptic LLPS-mediated protein complexes.

The current MS screening also identified many mitochondrial proteins as SUMO2/3 targets. The results are consistent with the presence of SUMOylation and deSUMOylation enzymes as well as SUMOylated proteins associated with mitochondria in cell line models or cultured neurons (Braschi et al., 2009; Akiyama et al., 2018; He et al., 2020). In addition, a number of studies dedicated to elucidate the function of specific mitochondrial SUMO targets indicated that SUMOylation participates in mitochondrial biogenesis (Guerra de Souza et al., 2016), regulates their fusion/fission processes (Guerra de Souza et al., 2016) and contributes to the metabolic adaption of mitochondria to stress (He et al., 2020). It is thus not surprising that SUMO2/3 substrates are also detected at synapses, as mitochondria are natural components of this compartment. Interestingly, mutations of the mitochondrial SUMO target DJ-1, also identified in the present screening, are associated with early-onset familial Parkinson’s disease (PD). The most deleterious DJ-1 mutation associated with PD leads to the aberrant SUMOylation of DJ-1 compromising the function and solubility of the protein (Guerra de Souza et al., 2016), which reinforced the idea that SUMOylation is essential to the synaptic mitochondrial function in physiological conditions.

The overall SUMO system is essential for the proper development and function of the brain. Indeed, disruption of the SUMOylation/deSUMOylation balance in animal models leads to deleterious effects such as neurodegeneration, seizure, impairments in learning and memory and/or dysfunctional behaviors (Henley et al., 2018; Huang et al., 2020; Yu et al., 2020). In the context of hypothermia, hibernation or ischemia, SUMOylation levels are significantly increased as part of a physiological neuroprotective response to stressful events. Thus, many efforts have been arrayed to pharmacologically modulate the SUMOylation machinery to protect the brain from damages caused by cerebral ischemia (Bernstock et al., 2018). Several studies have also highlighted a tight link between SUMO and brain disorders (Schorova and Martin, 2016; Anderson et al., 2017). In neurodegenerative disorders such as Alzheimer’s and Parkinson’s diseases, several proteins involved in the etiology of these diseases are modified by SUMO (Anderson et al., 2017). In addition, human AD brains present an increased SUMOylation level in both cortical tissue and synaptosomal lysates (Marcelli et al., 2017). Consistent with these findings, diseases annotation connects the present SUMO2/3 dataset to a large spectrum of neurological disorders, from neurodevelopmental pathologies to neurodegenerative diseases including schizophrenia, autism, epilepsy, Alzheimer’s and Parkinson’s diseases. By enlarging the repertoire of pathophysiological processes potentially affected by SUMOylation, our results provide additional perspectives to propose new biological markers or complementary therapeutic strategies. In particular, considering the neuroprotective effects of SUMO in response to cellular stresses, drugs modulating the SUMO system may be used to target brain pathologies involving altered SUMOylation levels.

To conclude, we report here that around 18% of the synaptic proteome is detected as SUMO2/3-ylated and propose that this post-translational modification regulates a wide range of cellular processes from synapse formation to synaptic transmission and plasticity. SUMO2/3 modifies many functionally connected synaptic proteins, suggesting that protein-group SUMOylation occurs at synapses and contributes to the modulation of synaptic multimeric complexes. A broader view would now require the characterization of the SUMO1-ylome as well as the identification of synaptic proteins presenting SUMO-interacting motifs. Moreover, SUMOylation evolving with brain development and life span, it would be particularly interesting to follow the SUMOylation profile of synaptic proteins over the age. With the current work, we finally propose an overall picture of the SUMO2/3 landscape at the mammalian synapse. Recent advances in imaging techniques allow to spatially and functionally map individual synapses according to their content in specific markers (Zhu et al., 2018) revealing that synapses are much more diverse than anticipated with each brain region having specific signatures regarding their synaptic composition. It is likely that PTM also participate in this identity card. In this context, detecting the SUMOylation status of specific target proteins in the brain at the single cell level is likely one of the most promising challenge of the future.

## Data Availability Statement

The raw data supporting the conclusions of this article will be made available by the authors, without undue reservation.

## Ethics Statement

The animal study was reviewed and approved by the Animal Care and Ethics Committee (Comité Institutionnel d’Ethique Pour l’Animal de Laboratoire N°28, Nice, France).

## Author Contributions

MP and CG performed the biochemical experiments with the help of GP and LS. FK, RF, and CG performed the bioinformatics. A-SG and DD performed the Mass Spectrometry analysis. MP, SM, and CG contributed to hypothesis development and data interpretation, and edited the manuscript. SM and CG provided the overall supervision and human resources. SM provided the funding. CG wrote the original draft. All authors discussed the data and commented on the manuscript.

## Conflict of Interest

The authors declare that the research was conducted in the absence of any commercial or financial relationships that could be construed as a potential conflict of interest.

## Publisher’s Note

All claims expressed in this article are solely those of the authors and do not necessarily represent those of their affiliated organizations, or those of the publisher, the editors and the reviewers. Any product that may be evaluated in this article, or claim that may be made by its manufacturer, is not guaranteed or endorsed by the publisher.
