# Supplementary material for: Proteomic Identification of an Endogenous Synaptic SUMOylome in the Developing Rat Brain
Source: Front Mol Neurosci. 2021 Nov 23;14:780535. doi: 10.3389/fnmol.2021.780535 (PMC8650717; doi:10.3389/fnmol.2021.780535)
Supplement: Supplementary file 7 [file Image_1.pdf]

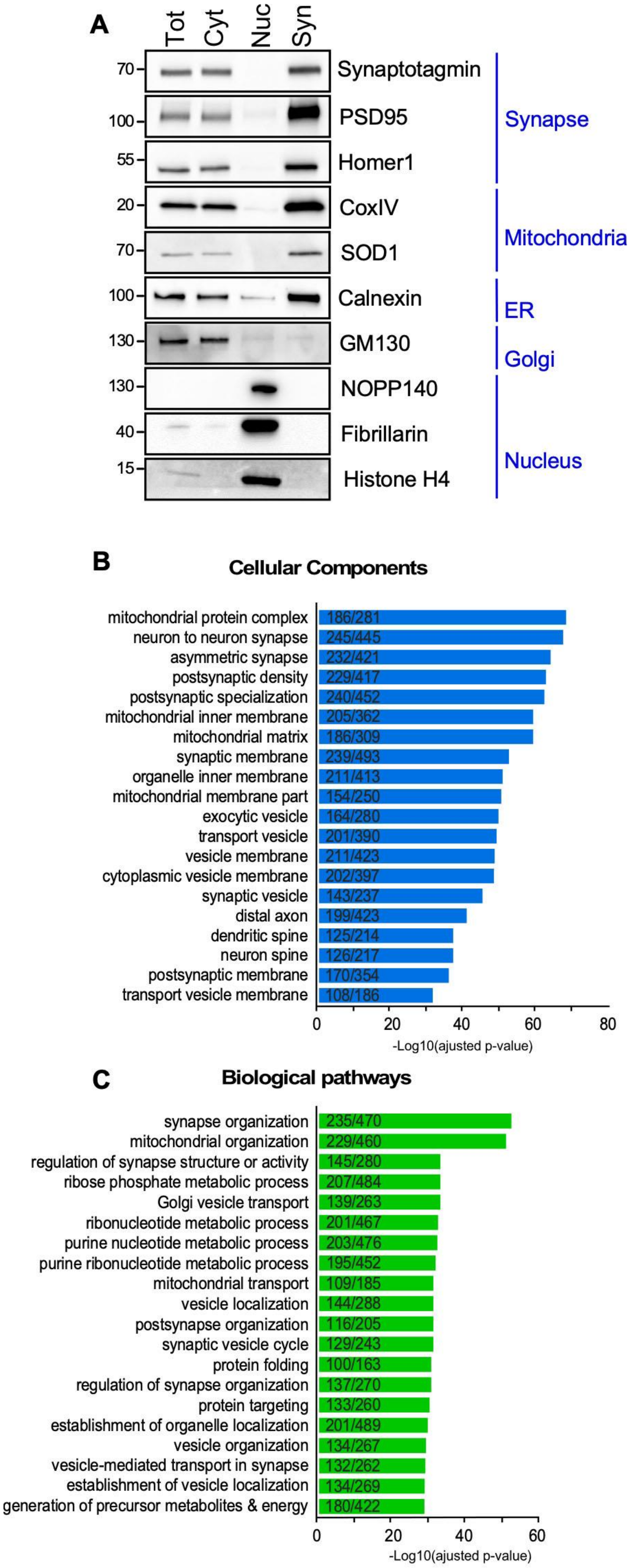

Supplementary Figure 1

Supplementary Figure 1: Quality control of the synaptic proteome. **A**. Western blot analysis of brain subcellular fractions. Tot = total brain lysate, Cyt = cytoplasmic fraction, Nuc = nuclear fraction, Syn = synaptosomal fraction. The list of the identified synaptic proteins was subjected to enrichment analysis for the GO Cellular Components terms (**B**) or the GO Biological Pathways terms (**C**) against the rat proteome. Categories were classified according to the -Log10 of the adjusted *p* value using the Benjamini-Hochberg method. The number of counts compared to the total number of hits in the category is indicated. Enrichment details are available in Supplementary Table 6.

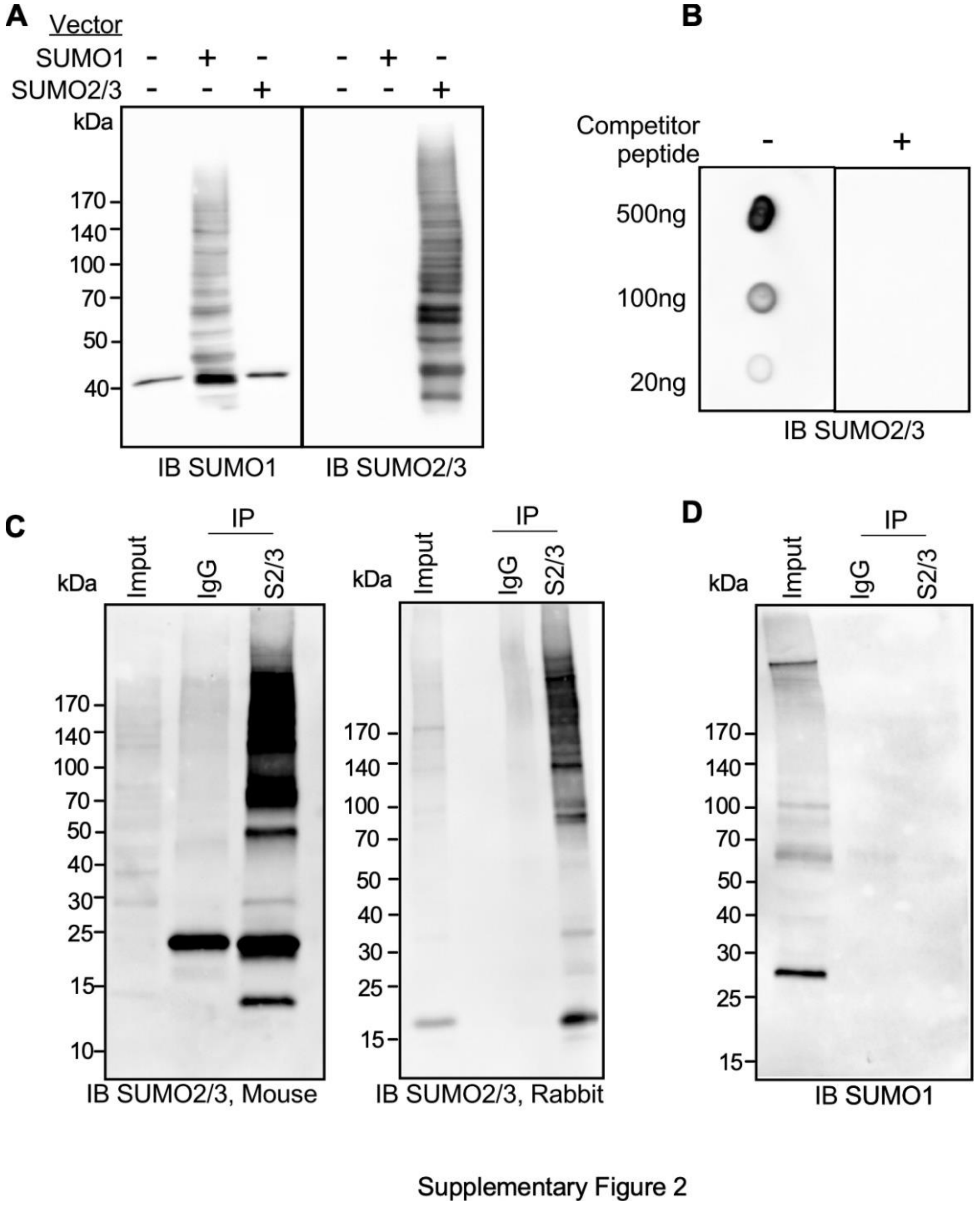

Supplementary Figure 2: **Characterization of the 12F3 mouse monoclonal SUMO2/3 antibody. A**. Western blotting on bacterial extract from Bl21 bacteria containing (+) or not (-) a SUMOylation system specifically expressing and conjugating SUMO1 or SUMO2. **B**. Dot blot performed on immobilized recombinant SUMO2 protein detected by SUMO2/3 12F3 antibody (dilution 1/2000) pre-incubated (right panel) or not (left panel) with the CQIRFRFDGQPINE peptide at final concentration of 10 μg/ml. **C-D**. Western blotting on SDS eluate from denaturing immunoprecipitation on whole PND14 rat brain extracts (input lane) using immobilized mouse IgG antibody (IgG lane) or 12F3 monoclonal mouse SUMO2/3 antibody (S2/3 lane). Immunopurified proteins (IP) were detected using the 12F3 mouse monoclonal SUMO2/3 antibody (**C left panel**), polyclonal rabbit SUMO2/3 (**C right panel**) or the 21C7 mouse monoclonal SUMO1 antibody (**D**).
